# New Pharmacological Strategies for the Treatment of Non-Infectious Uveitis. A Minireview

**DOI:** 10.3389/fphar.2020.00655

**Published:** 2020-05-08

**Authors:** Rodrigo A. Valenzuela, Iván Flores, Beatriz Urrutia, Francisca Fuentes, Pablo E. Sabat, Carolina Llanos, Loreto Cuitino, Cristhian A. Urzua

**Affiliations:** ^1^Laboratory of Ocular and Systemic Autoimmune Diseases, Faculty of Medicine, University of Chile, Santiago, Chile; ^2^Department of Chemical and Biological Sciences, Faculty of Health, Universidad Bernardo O Higgins, Santiago, Chile; ^3^Rheumatology Service, Department of Medicine, Hospital Clinico Universidad de Chile, Santiago, Chile; ^4^Department of Ophthalmology, University of Chile, Santiago, Chile; ^5^Department of Ophthalmology, Clínica las Condes, Santiago, Chile; ^6^Departamento de Inmunología Clínica y Reumatología, Escuela de Medicina, Pontificia Universidad Católica de Chile, Santiago, Chile; ^7^Servicio de Oftalmología, Hospital Clínico Universidad de Chile, Santiago, Chile; ^8^Faculty of Medicine, Clinica Alemana-Universidad del Desarrollo, Santiago, Chile

**Keywords:** uveitis, intraocular inflammation, biologics, anti-tumor necrosis factor, treatment

## Abstract

Non-infectious uveitis (NIU) is a group of disorders characterized by intraocular inflammation at different levels of the eye. NIU is a leading cause of irreversible blindness in working-age population in the developed world. The goal of uveitis treatment is to control inflammation, prevent recurrences, and preserve vision, as well as minimize the adverse effects of medications. Currently, the standard of care for NIU includes the administration of corticosteroids (CS) as first-line agents, but in some cases a more aggressive therapy is required. This includes synthetic immunosuppressants, such as antimetabolites (methotrexate, mycophenolate mofetil, and azathioprine), calcineurinic inhibitors (cyclosporine, tacrolimus), and alkylating agents (cyclophosphamide, chlorambucil). In those patients who become intolerant or refractory to CS and conventional immunosuppressive treatment, biologic agents have arisen as an effective therapy. Among the most evaluated treatments, TNF-α inhibitors, IL blockers, and anti-CD20 therapy have emerged. In this regard, anti-TNF agents (infliximab and adalimumab) have shown the strongest results in terms of favorable outcomes. In this review, we discuss latest evidence concerning to the effectiveness of biologic therapy, and present new therapeutic approaches directed against immune components as potential novel therapies for NIU.

## Introduction

Uveitis included a group of heterogeneous diseases characterized by inflammation of the uveal tract. It is an important cause of vision loss accounting for approximately 25% of total blindness in developed countries, particularly among the working-age population (Thorne et al., 2016). Uveitis may be generally classified by the etiology of inflammation as infectious or non-infectious (autoimmune disorders), which could be related or not to a systemic disease. If after a comprehensive investigation there is no a clear cause of inflammation, it is considered as idiopathic (Nussenblatt, 1990). The differential diagnosis for autoimmune uveitis includes systemic inflammatory diseases—such as spondyloarthropathies (SPA), inflammatory bowel disease (IBD), rheumatoid arthritis (RA), Behçet's disease (BD), sarcoidosis, or juvenile idiopathic arthritis (JIA)-, and autoimmune diseases that preferentially affect ocular tissues—such as Vogt-Koyanagi-Harada (VKH), white dot syndromes, sympathetic ophthalmia, and birdshot chorioretinopathy (BCR). In addition, uveitis can be anatomically classified as anterior, intermediate, posterior or panuveitis, and they may have an acute, chronic or recurrent course. The clinical presentation is variable, the symptoms may include blurred vision, photophobia, ocular pain and significant visual impairment (Keino et al., 2017). The etiology of uveitis depends on environmental, cultural, and genetic factors (Chan et al., 1985). In a recently published work by our group, uveitis was found to be secondary to a non-infectious autoimmune disease in 42% of cases. Infectious agents cause another 16.8%, while the condition remains idiopathic in the remaining 41% (Liberman et al., 2015).

### Pathophysiology of Uveitis

The pathophysiology understanding of intraocular inflammation is limited. The eye has immunological properties known as “immune privilege,” in which conventional immune response in not evoked by foreign antigens placed in those tissues. The haemato-ocular barriers, the lack of cell expression of both MHC-I and MHC-II, and the presence of immune modulators, such as TGF-β, among others, give this feature to the eye (Liberman et al., 2015).

It is thought that most non-infectious uveitis (NIU) are mediated by T cells, predominantly CD4+ T cells that exhibit a Th1 phenotype. Although the exact pathogenesis of uveitis is not completely understood, cytokines such as IL-2, TNF-α, INF-γ, IL-12, and IL-17 have been detected in patients with active disease, and it is believed that they would be key players in the pathogenesis (Durrani et al., 2004; Levy-Clarke et al., 2014; Dunn, 2015). Thus, these cytokines may be therapeutic targets for new treatments of NIU, whose management represents a challenge, given the broad variability in its nature and pathophysiology.

### Treatment of Non-Infectious Uveitis

The first line of treatment for NIU corresponds to corticosteroids (CS), particularly due to their high efficacy and rapid control of acute inflammation. Depending on the severity of the condition and the type of ocular involvement, CS can be administered topically, periocularly, intraocularly, or systemically (Poetker and Reh, 2010; Knickelbein et al., 2017; Rossi et al., 2019). However, systemic CS have been associated not only with a significant number of systemic adverse effects with long-term therapy—including diabetes, Cushing's syndrome, hypercholesterolemia, and osteoporosis—but also cataracts and glaucoma, which can ultimately result in visual impairment and blindness (Rothova et al., 1996; Poetker and Reh, 2010; Multicenter et al., 2011). The most studied and used CS is prednisolone, having the greatest clinical evidence supporting its use (Jabs et al., 2000; Biswas et al., 2004; Hedayatfar et al., 2014; Sheppard et al., 2014). In some clinical scenarios, immunosuppressant therapy (IMT)—such as antimetabolites (methotrexate, mycophenolate mofetil, azathioprine), calcineurinic inhibitors (cyclosporine, tacrolimus), and alkylating agents (cyclophosphamide, chlorambucil)—is required to treat uncontrolled inflammation (Knickelbein et al., 2017; Hassan et al., 2019; Rossi et al., 2019). In addition, these drugs are the standard of care in some clinical entities, such as BD, BCR, or pediatric uveitis, due to the higher prevalence of chronic disease (e.g., JIA-associated uveitis) in this last subset of patients (Knickelbein et al., 2017; Sood and Angeles-Han, 2017; Cann et al., 2018).

Biological therapy has emerged as a new therapeutic approach in pediatric and adulthood uveitis, based on targeting relevant immunological pathways involved in disease pathogenesis, particularly tumor necrosis factor alpha (TNF-α) (Knickelbein et al., 2017; Sood and Angeles-Han, 2017; Cann et al., 2018; Hassan et al., 2019).

### Anti TNF-α Drugs

TNF-α is a pro-inflammatory cytokine that participates in the pathophysiology of autoimmune and inflammatory diseases, and thus, it has become an important therapeutic target (Kuiper et al., 2017). TNF-α blockers have been used for the treatment of rheumatic diseases since 1999 and have dramatically changed the clinical course of invalidating diseases, such as RA and SPAs (Maini et al., 1999). Noteworthy, several trials and case series have already demonstrated the effectiveness of anti-TNF-α for treating refractory uveitis (**Table 1**) (Kruh et al., 2014; Vallet et al., 2016), showing a reduction of inflammatory signs and relapses, as well as an increase of the likelihood of visual acuity preservation (more than 90% in some cases) and the quality of life of uveitis patients (Fabiani et al., 2019a; Leal et al., 2019; Sharma et al., 2019). If uveitis is refractory to systemic CS and IMT or patients become intolerant to such therapies, a good sight-saving measure is the use of biological agents (Mercier et al., 2018; Trivedi and Katelaris, 2018).

**Table 1:** Current anti-TNF-α available treatments in non-infectious uveitis.

Therapy	Description	Study type	References
Adalimumab	Anti TNF-α humanized antibody	Clinical trial	(Diaz-Llopis et al., 2008)
		Systematic review	(Borrás-Blasco et al., 2015)
		Clinical trial	(Jaffe et al., 2016)
		Clinical trial	(Goto et al., 2019)
		Prospective study	(Sharma et al., 2019)
		Meta-analysis	(Leal et al., 2019)
		Systematic review	(Leal et al., 2018)
		Clinical trial	(Suhler et al., 2018)
		Clinical trial	(Lee et al., 2018)
		Retrospective study	(Mercier et al., 2018)
		Clinical trial	(Nguyen et al., 2016)
Infliximab	Anti TNF-α chimeric antibody	Systematic review	(Borrás-Blasco et al., 2015)
		Prospective study	(Sharma et al., 2019)
		Meta-analysis	(Leal et al., 2019)
		Systematic review	(Leal et al., 2018)
		Retrospective study	(Mercier et al., 2018)
Golimumab	Anti TNF-α humanized antibody	Systematic review	(Borrás-Blasco et al., 2015)
		Meta-analysis	(Leal et al., 2019)
		Systematic review	(Leal et al., 2018)
Etanercept	Anti TNF-α dimeric protein, act as a decoy for TNF-α Receptor	Meta-analysis	(Leal et al., 2019)
		Systematic review	(Leal et al., 2018)
Certolizumab	Anti TNF-α antibody	Systematic review	(Leal et al., 2019)
		Systematic review	(Leal et al., 2018)

TNF-α, tumor necrosis factor alpha.

#### Adalimumab

Adalimumab (Humira^®^) is an entirely humanized monoclonal antibody against TNF-α which is may be subcutaneously self-administered. It is the most used and studied biologic medication for the treatment of adulthood NIU since its approval in 2016 (Borrás-Blasco et al., 2015; Humira (R), (adalimumab), 2016).

The first report of adalimumab's role in the treatment of NIU was in 2008 (Diaz-Llopis et al., 2008). Subsequently, large multicenter randomized clinical trials (Visual I, II, and III) have showed this drug is effective in non-anterior NIU for inflammation control and as steroid-sparing agent (Diaz-Llopis et al., 2008; Jaffe et al., 2016; Nguyen et al., 2016; Leal et al., 2018; Lee et al., 2018; Suhler et al., 2018; Goto et al., 2019). The Visual I study was a multinational phase 3 trial where adalimumab given fortnightly, was used to treat active non-infectious intermediate, posterior, and panuveitis. The authors showed the rate of treatment failure was slashed to 50% in the adalimumab group compared with placebo (Jaffe et al., 2016). In the Visual II multicenter, randomized trial, adalimumab also lowered the risk of uveitis recurrence or vision loss, upon steroids withdrawal, in patients with inactive, non-infectious intermediate, posterior, and panuveitis, controlled by moderate systemic steroids dose. Treatment failure occurred in 55% of patients in the placebo group compared with 39% of patients in the adalimumab group (Nguyen et al., 2016). Noteworthy, although the good efficacy shown in these trials, further studies have found up to 40% of therapeutic failure after 12 months of treatment (Suhler et al., 2018).

In pediatric patients, adalimumab was approved in 2017 (Humira (R), (adalimumab), 2016; Shahab et al., 2018; Suhler et al., 2018). The combination of adalimumab with CS has been described as beneficial in terms of lowering therapeutic failure rate (Simonini et al., 2014). Ramanan et al. published a randomized trial that compared efficacy of adalimumab in children with active JIA-related uveitis despite methotrexate. In their work, patients assigned to the adalimumab arm had a significant lower treatment failure compared with placebo (27% vs 60%; *p* < 0.0001) (Ramanan et al., 2017). However, drug-induced remission of JIA-associated uveitis did not persist when the drug was withdrawn after 1 to 2 years of treatment (Horton et al., 2019). In addition, it has been showed that adalimumab is more effective for controlling inflammation and lowering relapses in pediatric NIU, in comparison with infliximab and etanercept (Simonini et al., 2011; Simonini et al., 2014).

Pediatric doses are started with a minimum of 24 mg/m^2^, and a maximum of 40 mg weekly. Adult scheme doses are started with a loading dose of 80 mg, and then maintenance of 40 mg every 2 weeks (Simonini et al., 2014; Sood and Angeles-Han, 2017).

Importantly, a bimodal agent of adalimumab (SB5) has been recently approved for the treatment of NIU and other autoimmune entities, such as RA, JIA, IBD, among others (Frampton, 2018).

#### Infliximab

Infliximab (Remicade^®^) is a chimeric monoclonal antibody used since 2001 (Sfikakis et al., 2001). It has 25% murine and 75% humanized domains. Its use is FDA-approved for RA, psoriatic arthritis (PsA), IBD, and AS, but not for NIU. It is only intravenously administered, usually in conjunction with methotrexate to prevent the generation of antibodies against the drug (Maini et al., 1999; Sood and Angeles-Han, 2017).

There is great evidence of its efficacy in NIU, mainly BD (Sfikakis et al., 2001; Vallet et al., 2016; Fabiani et al., 2018). Maleki et al., in a small retrospective case series, achieved remission in 19 of 23 patients with active intermediate NIU refractory to at least one IMT (Maleki et al., 2017). Along the same line, Baughman et al. showed a remarkable improvement in 13 out of 14 patients with several underlying causes of ocular inflammation, who were treated with infliximab after failure of classical IMT (Baughman et al., 2005) In other retrospective study, Bodaghi et al. achieved a rapid control of uveitis in all 12 patients refractory to CS and IMT (Bodaghi et al., 2005). Suhler et al. conducted a prospective non-comparative trial of infliximab therapy for refractory uveitis, in which 18 out of 23 patients met criteria for clinical success at week 10 (Suhler et al., 2005).

Nevertheless, despite these good results, some studies have indicated that the rate of therapeutic failure at 12 months of treatment is around 60% (Bodaghi et al., 2005; Simonini et al., 2011; Simonini et al., 2013a), which puts it at a disadvantage compared to adalimumab. However, infliximab presents a rapid onset of action, which is why it is recommended in severe exacerbations (Sfikakis et al., 2001; Markomichelakis et al., 2011).

The posology is very variable. In adults, the dose and frequency depend on the disease, which can be between 3 and 5 mg/kg every 6 to 8 weeks. The pediatric dose begins with a loading dose between 3 and 5 mg/kg at weeks 0, 2, and 6, and continues with a maintenance dose of at least 7.5 mg/kg/dose every 4 to 8 weeks; the dose is adjusted according to the clinical response and the patient's tolerance to the medication, with a evaluated maximum dose of 20 mg/kg (Sukumaran et al., 2012; Sood and Angeles-Han, 2017).

#### Golimumab

Golimumab (Simponi^®^) is a fully humanized monoclonal antibody, subcutaneously administered with a dose of 50 mg every 4 weeks. Its use has been approved for the treatment of AS, RA, PsA, and UC (Sukumaran et al., 2012; Calvo-Río et al., 2016). There is little evidence, but it has been described its efficacy in patients with NIU refractory to adalimumab or infliximab, and thus golimumab is usually reserved as treatment for this subset of non-responders (Miserocchi et al., 2014; Calvo-Río et al., 2016; Fabiani et al., 2019b). In that sense, this medication would present a higher affinity for the receptor, being able to improve clinical signs of inflammation in refractory patients (Tosi et al., 2019). Tosi et al. published a retrospective study of 21 uveitis patients treated with Golimumab (10 patients) or Certolizumab pegol (11 patients). They showed a significant reduction in ocular flares from 128.6 events/100 patients-year to 24.9 events/100 patients-years, although no specific results were published for each group. All patients in the Golimumab group had received another anti-TNF drug previously (Tosi et al., 2019). Additionally, Palmou-Fontana et al. showed complete remission of four of seven patients with JIA-associated uveitis treated with golimumab (Palmou-Fontana et al., 2018).

#### Etanercept

The mechanism of action of etanercept (Enbrel^®^) differs from the other anti-TNF-α agents. It is a dimeric protein that has an Fc portion of human IgG and a portion of the ligand-binding site of the TNF receptor p75 (Scott, 2005). Etanercept is subcutaneously administrated at a dose of 50 mg once per week or 25 mg twice a week. In children over 2 years old, etanercept is approved for the treatment of JIA at the dose of 0.4 mg/kg (up to a maximum of 25 mg/dose) twice a week or 0.8 mg/kg (maximum 50 mg) once weekly (Guillot et al., 2017).

There is little evidence about this medication, and case reports indicate that although patients with BD respond to therapy, this effect is not sustained (Simonini et al., 2014). Etanercept is no longer prescribed for uveitis given its low distribution in ocular tissue, and its lower efficacy compared to adalimumab and infliximab (Foster et al., 2003; Tynjälä et al., 2007; Touhami et al., 2019).

#### Certolizumab

Certolizumab (Cimzia^®^) is a recombinant humanized monoclonal antibody administered subcutaneously at an initial dose of 400 mg at weeks 0, 2, and 4 and then 200 mg per week. It is approved for the treatment of AS, PsA, RA, and Crohn's disease (Llorenç et al., 2016). It has been little studied in NIU, and in general, it has been evaluated in small case series. However, it has shown positive results, and no major adverse effects have been reported during its use (Rudwaleit et al., 2016; Lopalco et al., 2017a). It seems to have a better and higher distribution toward inflamed tissues than adalimumab and infliximab (Llorenç et al., 2016). A recent study has indicated that both golimumab and certolizumab represent effective and safe options for patients with uveitis even if the result with other anti-TNF-α drugs has not been successful (Tosi et al., 2019).

### Adverse Effects of Anti TNF-α Drugs

As adalimumab and golimumab are fully humanized antibodies, they have less immunogenic potential than chimeric antibodies (Simonini et al., 2014). The main adverse effects of anti-TNF-α agents include development of autoimmune diseases, increased risk of infection, where tuberculosis and histoplasmosis stand out, among others. Reactions at the injection site have been also described. In addition, anti-TNF-α blockers have been associated with an increased risk of malignancy, mainly lymphomas (Diak et al., 2010), although further studies have ruled this out (Shelton et al., 2016) and with debut or worsening of demyelinating disorders, such as multiple sclerosis (Magnano et al., 2004).

Adverse effects of adalimumab also include arthralgias, nasopharyngitis, and headaches. Side effects of infliximab may include the development of mild or severe lupus-like symptoms (9% and 1% respectively) (Levy-Clarke et al., 2014). Golimumab may cause nausea, fever, and anemia (Diak et al., 2010). In the case of etanercept, uveitis, IBD flares, and granulomatous diseases have been shown to be more prevalent compared to other TNF blockers (Scott, 2005; Guillot et al., 2017). Certolizumab has been little studied in NIU, and in general, it has been evaluated in small case series, showing positive results and mild or moderate adverse effects during its use (Rudwaleit et al., 2016; Lopalco et al., 2017b), with infections being the most common (Bykerk et al., 2015), and in fewer cases it has been reported a generalized skin reaction (in the third administration), anaphylactic reactions, and a lupus-like episodes (Bykerk et al., 2015).

A significant proportion of patients experiences loss of response during maintenance treatment with anti-TNF-α, due to the development of an immune response directed at the drug itself, leading to lower concentration of anti-TNF-α and the presence of anti-anti TNF-α antibodies in serum (Guerra et al., 2011; Touhami et al., 2019). The measurement of drug concentrations and probably, the measurement of anti-infliximab or anti-adalimumab antibodies, might be useful for improving the selection of patients who would benefit from the maintenance treatment with infliximab or adalimumab, avoiding inappropriate treatments, and helping to decide which procedure to follow in case of loss of response (Guerra et al., 2011). However, there is no enough evidence to recommend level or antibody measurements as standard of care in uveitis patients.

IMT are added in order to reduce the immunogenicity against anti-TNF-α drugs. Although there is no solid evidence-based data regarding their use, the use of an immunosuppressant together, such as methotrexate, is suggested in case of pathologies-associated uveitis (e.g. BD, JIA, etc.) (Touhami et al., 2019).

### Other Biologics Therapies

When conventional IMT and/or anti-TNF-α therapies fail, other biological agents are recommended. Some of the newest therapies have focused on blocking interleukin actions (**Table 2**).

**Table 2:** Current available non–anti-TNF Biologics treatments in non-infectious uveitis.

Therapy	Description	Study type	References
Tocilizumab	Anti-IL-6 humanized antibody	Systematic review	(Lopalco et al., 2017a)
		Clinical trial	(Papo et al., 2014)
		Systematic review	(Cann et al., 2018)
		Systematic review	(Sharma et al., 2018)
		Retrospective study	(Silpa-Archa et al., 2016)
Secukinumab	Anti-IL-17A monoclonal antibody	Clinical trial	(Dick et al., 2013)
		Review	(Sanford and McKeage, 2015)
Canakinumab	Anti-IL-1β	Case report	(Simonini et al., 2013b)
		Systematic review	(Simonini et al., 2015)
Anakinra	IL-1 receptor antagonist	Systematic review	(Simonini et al., 2015)
Rituximab	Anti CD20 antibody	Systematic review	(Simonini et al., 2015)
		Retrospective study	(Miserocchi et al., 2016)
		Clinical trial	(Davatchi et al., 2010)

IL, interleukin; CD, cluster of differentiation.

### Anti-IL-6 Therapies

Interleukin-6 (IL-6) is a proinflammatory cytokine implicated in various immune-mediated diseases. Its produced by monocytes, macrophages, B cells, and T cells mainly. In some pathologies, such as central retinal vein occlusion, diabetes, or uveitis, intraocular concentrations of IL-6 are increased (Petrinović-Doresić et al., 1999). The recombinant human monoclonal antibody tocilizumab (Actemra^®^) is an IL-6 receptor blocker and has shown a good effect in the treatment of NIU (Lopalco et al., 2017a), particularly in BD patients with refractory uveitis (Atienza-Mateo et al., 2018). FDA approved the use of tocilizumab for the management of RA, including refractory patients (Hassan et al., 2019). In general, tocilizumab, presents minor adverse effects such as chest tightness, fatigue, nausea, and blisters on the hands and limbs (Silpa-Archa et al., 2016; Sharma et al., 2018), non-serious bronchitis, leukopenia, and thrombocytopenia in which the drug was not suspended (Papo et al., 2014). However, cases with more severe manifestations such as neutropenia, unacceptable dizziness and nausea, severe infusion reaction, severe angioedema, and severe abdominal pain have been reported, requiring discontinuation of the medication (Silpa-Archa et al., 2016; Atienza-Mateo et al., 2018). There is a reported case of probable association of anxiety, sweating, and flushing with the infusion (Sepah et al., 2017).

#### Anti-IL-1 Therapies

Anti-IL-1 treatment includes antagonist of the IL-1 receptor, anakinra (Kineret^®^), and anti-IL-1β antibody, canakinumab (Ilaris^®^). Both anti-IL-1s were evaluated in a multicenter retrospective study that included 19 BD-associated uveitis patients. In this study, both treatments were effective in the management of uveitis, providing a good control of inflammation and steroid-sparing effect, in refractory and long-lasting cases (Fabiani et al., 2017). IL-1 blockers have shown mild adverse effects, confirming their excellent safety profile (Fabiani et al., 2017). In the case of anakinra the most reported manifestations are headaches and arthralgias (Kullenberg et al., 2016; Cavalli and Dinarello, 2018). There is only a reported case of reactivation of pulmonary TB, but in an elderly patient with RA and record of pulmonary TB (Cavalli and Dinarello, 2018).

#### Anti-CD20 Therapies

Rituximab (Rituxan^®^/MabThera^®^) is a chimeric (human and murine portion) monoclonal antibody directed against the CD20 antigen present on the B-cells surface. In 1997 it was approved to treat lymphoma, and its use has continued to control other diseases, such as RA (Davatchi et al., 2010). In a retrospective study with JIA-related uveitis patients, all achieved complete control of uveitis within 5 months of first infusion over a mean follow up of 44 months, demonstrating the long-term efficacy of rituximab for anti-TNF-α refractory cases (Miserocchi et al., 2016). In addition, rituximab was effective for treating resistant ANCA-positive vasculitis, with no significant adverse effects (Eriksson, 2005). Regarding adverse effects, there are minor cases of hives and flushing, and others more severe that have required the suspension of the infusion. Infection-like pneumonia and herpes zoster have been also reported (Davatchi et al., 2010).

#### Anti-CD25 Therapies

Daclizumab is a monoclonal antibody targeted to 2α subunit of the IL-2 receptor on activated T-cells, blocking the conventional T-cells immune responses (Sen et al., 2009; Giovannoni et al., 2016). In this case, mild adverse effects are more frequent than serious. The most frequently reported adverse effects have been on the one hand herpes zoster, rash, and palpitations, and the other liver disorders and elevated transaminases, although mainly asymptomatic and self-limited (Sen et al., 2009; Giovannoni et al., 2016).

#### Anti-IL-17A Therapies

Secukinumab is a monoclonal antibody (fully humanized) that binds and neutralizes the IL-17A (Dick et al., 2013; Sanford and McKeage, 2015). Some problems in the upper respiratory tract (nasopharyngitis) and headache has been reported as adverse effects, and it has been seen cases of reactivation of uveitis, as well as arthralgias (Dick et al., 2013).

## Conclusions

Biologic agents have revolutionized the immunological treatment of NIU in the past 20 years. The most newly published guidelines for uveitis management suggest a step-ladder approach, starting with topical, periocular, and systemic CS, followed by IMT, and finally the use of biologic therapy (preferably a TNF-α inhibitor). In this sense, despite the well-known side effects of CS, they remain as the cornerstone of treatment for acute diseases and exacerbations. Furthermore, the use of new therapies is still limited due to their high cost, and all the aspects that remain to be studied, such as follow-up regimens and monitoring. Nevertheless, it would not be a surprise that, with the availability of new data, biological agents may be recommended as first-line therapy in the management of some causes of uveitis. Furthermore, new insights have emerged to present new therapeutic approaches directed against immune components as potential novel therapies for NIU.

## Author Contributions

RV and IF wrote the manuscript. RV and FF made the tables. RV, IF, BU, FF, PS, CL, LC, and CU read, discussed, and revised the manuscript. All authors listed have made a substantial, direct and intellectual contribution to the work and approved it for publication.

## Funding

This study was supported by Fondo de Fomento al Desarrollo Científico y Tecnológico (FONDEF) grant No. IT17I0087 (to CU) and National Agency for Research and Development (ANID) grant Fondecyt de Iniciación en Investigación No. 11191215 (to LC).

## Conflict of Interest

The authors declare that the research was conducted in the absence of any commercial or financial relationships that could be construed as a potential conflict of interest.
